# Challenges and Opportunities in Developing Tailored Pain Management Strategies for Liver Patients

**DOI:** 10.7759/cureus.50633

**Published:** 2023-12-16

**Authors:** Manahil Majid, Muhammad Yahya, Frank Ansah Owusu, Saira Bano, Taha Tariq, Iqra Habib, Beesham Kumar, Maham Kashif, Giustino Varrassi, Mahima Khatri, Satesh Kumar, Arham Iqbal, Alina S Khan

**Affiliations:** 1 General Medicine, Diana Princess of Wales Hospital, Grimsby, GBR; 2 Medicine, Liaquat University of Medical and Health Sciences, Jamshoro, PAK; 3 Medicine, Stavropol State Medical University, St. Louis, USA; 4 Medicine, Faisalabad Medical College and University, Faisalabad, PAK; 5 Medicine, Lahore Medical and Dental College, Lahore, PAK; 6 Medicine, Mohtarma Benazir Bhutto Shaheed Medical College, Mirpur, PAK; 7 Medicine, Jinnah Medical and Dental College, Karachi, PAK; 8 Medicine, Khawaja Muhammad Safdar Medical Collge, Sialkot, PAK; 9 Pain Medicine, Paolo Procacci Foundation, Rome, ITA; 10 Internal Medicine/Cardiology, Dow University of Health Sciences, Civil Hospital Karachi, Karachi, PAK; 11 Medicine, Shaheed Mohtarma Benazir Bhutto Medical College, Karachi, PAK; 12 Medicine and Surgery, Dow University of Health Sciences, Dow International Medical College, Karachi, PAK; 13 Medicine, Liaquat National Hospital and Medical College, Karachi, PAK

**Keywords:** interdisciplinary collaboration, precision medicine, chronic liver conditions, tailored interventions, pain management, liver diseases

## Abstract

Chronic liver illnesses pose a substantial worldwide health challenge, with various causes that span from viral infections to metabolic problems. Individuals suffering from liver problems frequently face distinct difficulties in pain control, requiring a customized strategy that takes into account both the fundamental disease and the complexities of liver function. The liver, a vital organ responsible for metabolic control and detoxification, is pivotal in multiple physiological processes. Chronic liver illnesses, such as cirrhosis and non-alcoholic fatty liver disease (NAFLD), are marked by a gradual process of inflammation and fibrosis, resulting in reduced liver function. These disorders often come with pain, varying from internal discomfort to intense abdominal pain, which impacts the quality of life and general well-being of patients.

The review explores the complex aspects of pain perception in liver illnesses, including inflammation, modified neuronal signaling, and the influence of comorbidities. It highlights the significance of a detailed comprehension of the pain experience in individuals with hepatic conditions for the implementation of successful pain management treatments. In addition, the review emphasizes the difficulties involved in treating pain in this group of patients, such as the possible complications linked to commonly prescribed pain relievers and the necessity for collaboration between hepatologists, pain specialists, and other healthcare professionals. Moreover, it examines new possibilities in the domain, such as the significance of innovative pharmacological substances, non-pharmacological treatments, and personalized medicine strategies designed for specific patient characteristics. This study thoroughly analyzes the difficulties and possibilities involved in creating personalized pain management approaches for individuals with liver conditions. Its purpose is to guide physicians, researchers, and healthcare providers, enabling them to implement more efficient and patient-focused interventions. As our comprehension of liver-related pain progresses, the potential for enhancing the quality of life for persons with chronic liver disorders through tailored pain management measures becomes more and more encouraging.

## Introduction and background

Chronic liver diseases (CLDs) represent a formidable global health challenge, affecting millions of individuals and imposing a substantial burden on healthcare systems worldwide. Defined by a spectrum of conditions ranging from viral hepatitis to metabolic disorders like non-alcoholic fatty liver disease (NAFLD), CLDs progressively lead to liver inflammation, fibrosis, and impaired function. The staggering prevalence of CLDs necessitates a holistic understanding of their impact, extending beyond the traditional realms of hepatology. One pivotal aspect that often stands out in the lived experience of individuals grappling with CLDs is the presence of persistent pain, casting a shadow on their quality of life and overall well-being. As we explore challenges and opportunities in developing tailored pain management strategies for liver patients, it is crucial to grasp the vastness of the global burden posed by CLDs. 

An estimated 844 million people were affected by CLDs in 2019, with a staggering two million succumbing to liver-related complications annually [1]. Viral hepatitis, alcohol-related liver diseases, and NAFLD are among the leading contributors to this growing epidemic, underlining the urgent need for comprehensive management strategies. Within the intricate tapestry of CLDs, the prevalence of pain emerges as a profound and often underestimated aspect of the patient experience. Pain in liver diseases can manifest in various forms, from the dull ache associated with fibrosis to the intense abdominal discomfort experienced in advanced stages such as cirrhosis. The impact of pain extends beyond the physical realm, permeating into the emotional and psychological dimensions of patients' lives. Understanding the significance of pain in liver patients requires a multifaceted approach, acknowledging the physiological mechanisms and the psychosocial intricacies involved. Chronic pain not only compromises the patient's ability to perform daily activities but also contributes to mental health challenges, including anxiety and depression [2]. Furthermore, the persistent nature of pain can result in a diminished quality of life, making effective pain management a crucial component of holistic care for individuals with CLDs.

Amidst the landscape of pain management, a critical paradigm shift is underway, one that recognizes the need for tailored strategies customized to the unique challenges posed by liver diseases. Conventional approaches to pain management, often rooted in general principles, may fall short of addressing the specific nuances associated with CLDs. Tailoring interventions to the individual characteristics of liver patients becomes imperative, considering factors such as the underlying etiology of liver disease, the severity of liver damage, and the presence of comorbid conditions [2]. Precision in pain management involves not only the selection of appropriate pharmacological agents but also the incorporation of non-pharmacological interventions and a patient-centered approach to care.

The advent of precision medicine has opened new avenues for understanding the interplay between genetic factors, environmental influences, and the response to pain medications in liver patients. By deciphering these intricate relationships, healthcare providers can optimize pain management strategies, minimizing adverse effects and maximizing efficacy [3]. Beyond the scientific and clinical dimensions, the journey of pain management in liver patients is inherently human. Each traverses a unique path, shaped by personal experiences, cultural influences, and social contexts. Recognizing the human aspect of pain in CLDs is not only empathetic but also essential for tailoring interventions that resonate with patients' individual needs. The significance of a patient-centered approach cannot be overstated. A study by Hauser et al. demonstrated that incorporating patient perspectives into pain management decision-making not only improves treatment outcomes but also fosters a sense of empowerment and engagement in the healthcare process [4]. By understanding the lived experiences of liver patients, healthcare providers can create tailored interventions that align with patients' values, preferences, and goals.

As we navigate the intricacies of challenges and opportunities in developing tailored pain management strategies for liver patients, we must ground our exploration in the rich tapestry of human experiences. This narrative review aims not only to elucidate the scientific complexities but also to weave a narrative that resonates with the diverse and profound stories of individuals facing the dual challenges of CLDs and persistent pain. In the subsequent sections, we will delve into the pathophysiology of pain in liver diseases, the inherent difficulties in pain management, and the emerging opportunities that hold promise for a future where pain relief is effective and uniquely tailored to each patient's needs. In summary, CLDs present a global health crisis, with pain emerging as a poignant and multifaceted aspect of the patient experience. Recognizing the significance of pain in liver patients and understanding the imperative for tailored strategies sets the stage for a comprehensive exploration of the challenges and opportunities in this crucial healthcare domain. By embracing the human touch in pain management, we unravel the complexities, bridging the gap between scientific understanding and the lived experiences of individuals facing the dual burden of liver diseases and persistent pain.

## Review

Methodology

Conducting a narrative review on the challenges and opportunities in developing tailored pain management strategies for liver patients involves a comprehensive approach to gathering relevant information. The first step in our methodology involved a thorough search of scientific databases, including PubMed, Scopus, and Cochrane Library. Keywords such as "chronic liver diseases," "pain management," and "tailored strategies" were employed to identify articles published up to the knowledge cutoff date in November 2023. In addition to electronic databases, manual searches were conducted through relevant journals, textbooks, and grey literature to ensure a comprehensive understanding of the current landscape of pain management in liver patients. Articles selected for review focused on CLDs, pain management strategies, and the customization of interventions. Studies addressing challenges and opportunities in tailoring pain relief for liver patients were prioritized. Information was synthesized in a narrative format, organizing findings into coherent themes related to the pathophysiology of pain in liver diseases, current pharmacological and non-pharmacological interventions, and advancements in precision medicine. Given the nature of a narrative review, ethical approval was not required. However, strict adherence to the principles of transparency, accuracy, and respect for intellectual property rights guided the presentation of information. Acknowledging the inherent limitations of narrative reviews, including potential publication bias and subjectivity in data synthesis, we strived to maintain rigor through systematic search strategies and critical appraisal of included studies. However, readers should interpret the findings in light of these inherent limitations.

Pathophysiology of liver diseases and pain

CLDs cast a profound shadow on the health of millions worldwide, ushering in a cascade of challenges that extend beyond impaired liver function. Among the intricate facets of CLDs, pain emerges as a significant and often overlooked aspect, influencing the well-being and quality of life of affected individuals. This essay delves into the pathophysiology of liver diseases and pain, unraveling the mechanisms that underlie the often debilitating experience of pain in this patient population.

Mechanisms of Pain in Chronic Liver Conditions

Pain in chronic liver conditions is a complex phenomenon with multifactorial origins. Unlike acute pain, which serves as a warning signal in response to tissue injury, chronic pain in liver diseases often lingers and becomes a persistent companion to the affected individuals. The mechanisms driving this chronic pain are intricately woven into the fabric of liver pathophysiology. A crucial contributor to pain in chronic liver conditions is the distortion of liver architecture due to ongoing inflammation and fibrosis. As inflammation takes root within the liver parenchyma, it creates a cascade of events that ultimately leads to tissue scarring or fibrosis. This distorted tissue architecture contributes to the mechanical strain on the liver capsule, a sensitive structure surrounding the liver, resulting in a dull, persistent ache [5]. Moreover, the inflammatory milieu within the liver creates an environment rich in mediators such as cytokines and chemokines. These biochemical signals contribute to the activation of immune cells and sensitize nerve endings, heightening their responsiveness to stimuli and lowering the pain threshold [5].

In essence, the inflammatory processes within the liver create a fertile ground for the genesis and perpetuation of pain. Inflammation, a cornerstone of liver diseases, plays a dual role in pain perception. On one hand, it directly stimulates nerve endings within the liver, contributing to the sensation of pain. On the other hand, inflammatory mediators released during the immune response can sensitize the central nervous system, amplifying pain perception even without direct liver stimulation. Fibrosis, the progressive scarring of liver tissue, further exacerbates the pain experienced in chronic liver conditions. As the liver undergoes architectural changes, the once-supple organ becomes rigid and less compliant. This rigidity can lead to increased pressure within the liver, affecting the surrounding structures and triggering pain signals. Additionally, fibrosis disrupts the normal blood flow within the liver, contributing to congestion and ischemia, both recognized sources of pain [6]. The impact of inflammation and fibrosis on pain perception is not confined to the liver alone; it extends its tendrils into neighboring structures. The liver is richly innervated, and the pain experienced may radiate to the right upper quadrant, abdomen, or even the back. This referred pain phenomenon further complicates the clinical picture, making pinpointing the exact source of discomfort challenging.

Neural Signaling Alterations in Liver Diseases

Neural signaling alterations represent another layer in the intricate interplay between liver diseases and pain. The nervous system undergoes adaptive changes in response to the persistent inflammatory milieu and structural alterations within the liver. The peripheral nerves that innervate the liver, known as hepatic nerves, become hypersensitive in CLDs. This heightened sensitivity is fueled by the release of neurotransmitters such as substance P and calcitonin gene-related peptide (CGRP) from nerve endings. These substances not only contribute to the transmission of pain signals but also play a role in promoting inflammation within the liver [6]. The brain processes and interprets pain signals at the central nervous system level. In chronic liver conditions, maladaptive changes occur in the central nervous system, leading to a phenomenon known as central sensitization. This process involves amplifying pain signals, making individuals more susceptible to experiencing pain even with mild stimuli. Central sensitization intensifies the perception of pain originating from the liver and contributes to the development of widespread pain and hypersensitivity [7]. The pain mechanisms in CLDs are not isolated events but interconnected processes reflecting the intricate dance between inflammation, fibrosis, and neural signaling alterations. As we decipher the scientific underpinnings of pain in liver diseases, we must recognize that these mechanisms are not merely academic concepts but tangible experiences for individuals grappling with these conditions. The persistent ache, the nuanced discomfort, and the challenges posed by referred pain are not abstract; they are part of the daily reality for those navigating the labyrinth of CLDs.

Challenges in pain management for liver patients

CLDs bring with them a myriad of challenges, and one of the most pervasive is the persistent presence of pain. As we embark on a journey to understand the difficulties in pain management for liver patients, it becomes apparent that the intricate interplay between liver pathophysiology and pain perception presents unique hurdles. This essay explores the common pain challenges faced by liver patients, delves into the complications associated with traditional analgesics, and highlights the crucial considerations in the context of comorbidities and medication interactions.

Overview of Common Pain Challenges Faced by Liver Patients

Pain, in the context of CLDs, manifests in diverse forms, casting a broad shadow on the lives of affected individuals. From the dull, persistent ache associated with fibrosis to the more intense abdominal pain witnessed in advanced stages like cirrhosis, the spectrum of pain challenges faced by liver patients is extensive. A notable challenge lies in accurately assessing and characterizing the pain experienced by liver patients. The nature of liver pain is often visceral, meaning it originates from the internal organs, making it inherently challenging to localize and describe. Patients may use terms like heaviness, pressure, or discomfort rather than sharp or stabbing sensations commonly associated with other types of pain [7]. Liver pain's subjective and elusive nature complicates the diagnostic and management landscape. Moreover, the experience of pain is not uniform across liver patients. Factors such as the underlying etiology of liver disease, the extent of liver damage, and the presence of comorbidities contribute to the heterogeneity in pain presentation. Tailoring interventions to meet each patient's unique needs becomes paramount, necessitating a personalized approach to pain management.

In the pursuit of alleviating pain in liver patients, healthcare providers are often confronted with the challenge of selecting appropriate analgesic agents. Traditional analgesics, such as nonsteroidal anti-inflammatory drugs (NSAIDs) and opioid medications, pose significant complications in the context of liver diseases. NSAIDs, commonly used for pain relief and anti-inflammatory effects, exert their actions by inhibiting enzymes involved in the synthesis of prostaglandins. However, this mechanism has a notable drawback in the context of liver diseases. The liver, already compromised in function, may struggle to metabolize NSAIDs, leading to an increased risk of hepatotoxicity [8]. Individuals with cirrhosis, in particular, face heightened susceptibility to complications associated with NSAID use. The impaired liver function compromises the clearance of these medications, amplifying the risk of adverse effects, including gastrointestinal bleeding and acute liver injury [8]. As such, the seemingly innocuous choice of an over-the-counter pain reliever becomes a delicate balancing act in the context of liver patients. Opioids, potent analgesics widely used for pain management, present another layer of complexity in liver patients. While opioids may offer effective relief, their use raises concerns about the potential for exacerbating underlying liver conditions and contributing to complications such as hepatic encephalopathy [9]. Furthermore, liver patients may exhibit altered drug metabolism, affecting the pharmacokinetics of opioids. This altered metabolism, coupled with the potential for drug interactions, necessitates cautious prescribing and close monitoring to prevent unintended consequences. The opioid crisis and concerns related to addiction further complicate the landscape of opioid use in liver patients. Striking a balance between providing adequate pain relief and minimizing the risk of opioid-related adverse effects poses a substantial challenge for healthcare providers.

Considerations for Comorbidities and Medication Interactions

Beyond the complexities associated with analgesic choices, the challenges in pain management for liver patients extend to considerations of comorbidities and potential interactions with other medications. Liver patients often grapple with multimorbidity, meaning they contend with the presence of multiple chronic conditions simultaneously. The challenge lies not only in managing liver-related pain but also in addressing the intricacies introduced by comorbidities such as diabetes, cardiovascular disease, or renal impairment [10]. Each comorbidity adds a layer of complexity to the overall care plan, requiring a nuanced understanding of the interplay between different conditions and their respective treatments. Healthcare providers must navigate a delicate balance, ensuring that interventions for pain management do not exacerbate other chronic conditions while still addressing the unique challenges posed by liver diseases. Liver patients often find themselves on many medications to manage various aspects of their health. The potential for drug interactions, where one pill influences the effectiveness or safety of another, becomes a critical consideration in pain management. For example, medications metabolized by the liver may experience altered clearance in the context of impaired liver function. This altered metabolism can lead to drug accumulation, increasing the risk of adverse effects. Moreover, medications with similar metabolism pathways may compete for the limited enzymatic resources within the compromised liver, further complicating the pharmacokinetic landscape [10]. Anticipating and managing these potential interactions requires a comprehensive understanding of the patient's medication regimen, emphasizing the importance of communication and collaboration among healthcare providers involved in the patient's care.

Patient-centered pain assessment

Pain, a multifaceted and subjective experience, weaves itself into the fabric of the human condition. For individuals grappling with CLDs, pain becomes an integral part of their daily narrative. This essay explores patient-centered pain assessment, delving into the importance of personalized approaches, examining tools and methods tailored for evaluating pain in liver patients, and addressing the intricate psychosocial aspects that shape the pain experience.

Importance of Personalized Pain Assessment

At the heart of patient-centered care lies the recognition that individuals are not merely carriers of diseases; they are unique beings with distinct experiences, values, and aspirations. This recognition is particularly crucial when it comes to pain assessment in the context of CLDs. Pain is inherently subjective, varying between individuals and within the same person over time. Adopting a standardized approach to pain assessment often falls short of capturing the nuances of the pain experience in liver patients. A numerical rating on a pain scale may convey the intensity but falls short of encapsulating the quality, impact on daily life, and the emotional toll of pain. A personalized approach to pain assessment acknowledges the uniqueness of each patient's pain experience. Cultural background, personal beliefs, and coping mechanisms are pivotal in shaping how pain is perceived and expressed [10]. Tailoring assessments to align with these individual nuances fosters a deeper understanding of the patient's experience, laying the foundation for targeted interventions that resonate with their values and goals.

In pain assessment for liver patients, employing tools and methods that capture the multidimensional nature of pain becomes imperative. Traditional measures, such as the Visual Analog Scale (VAS) or Numeric Rating Scale (NRS), provide a quantitative snapshot of pain intensity but may fall short of encapsulating the broader aspects of the pain experience. Comprehensive pain inventories offer a more holistic approach to pain assessment, delving into the qualitative aspects of pain. The McGill Pain Questionnaire (MPQ) and the Brief Pain Inventory (BPI) are examples of tools that go beyond a numerical rating, capturing the sensory and affective components of pain [11]. For liver patients, whose pain may manifest in various forms, employing such comprehensive tools allows for a more nuanced understanding. Patient-reported outcomes (PROs) play a pivotal role in patient-centered pain assessment. By directly soliciting the patient's perspective, PROs provide insights into the impact of pain on daily functioning, emotional well-being, and overall quality of life. The use of PROs, such as the Pain Impact Questionnaire (PIQ-6) or the Patient-Reported Outcomes Measurement Information System (PROMIS), empowers patients to participate in their care actively and ensures that assessments align with their priorities [11].

Dynamic Assessments: Pain Diaries and Ecological Momentary Assessment (EMA)

Recognizing the dynamic nature of pain, especially in chronic conditions, calls for assessments that capture fluctuations over time. Pain diaries, where patients record their pain experiences regularly, offer a longitudinal perspective, allowing healthcare providers to discern patterns and triggers. Using mobile technology, ecological momentary assessment (EMA) enables real-time data collection, providing an in-the-moment snapshot of pain experiences in the natural environment [11]. Pain is not confined to the physical realm; it intertwines with psychosocial dimensions, influencing emotions, relationships, and overall well-being. A patient-centered approach to pain assessment in liver patients necessitates an exploration of these psychosocial aspects. Chronic pain often coexists with emotional challenges, including anxiety and depression. Understanding the dynamic landscape is essential, as it influences the perception of pain and shapes the individual's ability to cope. Incorporating validated tools for assessing emotional well-being, such as the Hospital Anxiety and Depression Scale (HADS), alongside pain assessments provides a more comprehensive view of the patient's mental health [11].

The significance of social support cannot be overstated in the context of chronic pain. Exploring the patient's support network, assessing coping mechanisms, and understanding how pain impacts relationships offer valuable insights. Tools like the Coping Strategies Questionnaire (CSQ) and the Multidimensional Scale of Perceived Social Support (MSPSS) provide avenues for probing into these psychosocial dimensions [12]. Cultural influences shape the expression and interpretation of pain. A patient's cultural background plays a pivotal role in how they communicate pain, their expectations regarding pain management, and the impact of pain on their identity. Embracing cultural humility and employing culturally sensitive tools, such as the Cross-Cultural Pain Questionnaire (CCPQ), ensures that pain assessments are conducted with cultural competence [12]. In the symphony of patient-centered care, pain assessment emerges as a poignant movement that harmonizes the scientific precision of validated tools with the nuanced understanding of individual narratives. The tools and methods employed should not be mere instruments; they should be conduits for capturing the lived experiences of liver patients grappling with the dual burden of chronic diseases and persistent pain. By embracing the importance of personalized approaches, incorporating comprehensive tools, and delving into the psychosocial dimensions of pain, healthcare providers can compose a melody that resonates with the uniqueness of each patient. As we navigate the landscape of pain assessment for liver patients, we decode the language of pain and empower individuals to articulate their experiences, fostering a therapeutic alliance grounded in empathy and understanding.

Current pharmacological interventions

Pain, an unwelcome companion in the journey of CLDs, demands a nuanced approach to intervention. This essay embarks on a detailed exploration of current pharmacological interventions for liver-related pain, traversing the landscape of existing analgesic options, critically examining their limitations and potential risks, and shedding light on emerging pharmaceutical approaches that hold promise in reshaping the narrative of pain management.

Review of Existing Analgesic Options

The pharmacological arsenal for managing liver-related pain encompasses a spectrum of options, each with its unique mechanisms of action and considerations. Understanding the landscape of existing analgesic options is crucial for healthcare providers navigating the complexities of pain management in the context of CLDs. Acetaminophen, often considered a first-line analgesic, presents a paradox in the realm of liver diseases. While it effectively alleviates pain and reduces fever, its metabolism occurs predominantly in the liver. In cases of compromised liver function, as seen in CLDs, the risk of hepatotoxicity escalates. The narrow therapeutic window of acetaminophen poses a challenge, requiring meticulous dosing and close monitoring to avert potential harm [13]. NSAIDs, with their potent anti-inflammatory and analgesic properties, are commonly utilized for pain relief.

However, their use in liver patients is riddled with complexities. The risk of gastrointestinal bleeding, coupled with the potential to exacerbate renal dysfunction and induce hepatorenal syndrome, necessitates caution [13]. In particular, individuals with cirrhosis face an elevated risk of complications associated with NSAID use, emphasizing the need for vigilant risk-benefit assessments. Opioids, potent analgesics central to pain management, present a delicate balance between providing relief and navigating potential complications. The liver, a hub for drug metabolism, plays a pivotal role in opioid processing. In the context of CLDs, alterations in drug metabolism may lead to opioid accumulation, raising concerns about respiratory depression and other opioid-related adverse effects [13]. Furthermore, the opioid epidemic underscores the imperative to approach opioid use judiciously, considering the risk of addiction and the potential for unintended consequences. Striking a balance between adequate pain relief and minimizing the risk of opioid-related complications demands a personalized and vigilant approach. Beyond conventional analgesics, adjuvant medications play a role in addressing neuropathic pain, a common dimension of liver-related pain. Drugs such as gabapentin and pregabalin, designed initially to manage seizures, exhibit efficacy in dampening neuropathic pain signals. However, their use requires careful titration and monitoring due to potential side effects, including sedation and dizziness [14].

Limitations and Potential Risks Associated with Standard Medications

While existing analgesic options provide a foundation for pain management in liver patients, their utilization is fraught with limitations and potential risks intrinsic to the intricate interplay between liver function and drug metabolism. Hepatotoxicity, a common thread woven through many analgesic options, poses a substantial stake in the context of CLDs. Acetaminophen, despite its efficacy, becomes a potential culprit in liver injury, especially when consumed at doses exceeding recommended limits. NSAIDs, while offering anti-inflammatory benefits, may contribute to liver damage, particularly in individuals with advanced liver disease [14]. Opioids, with their metabolism intricately linked to liver function, pose a dual risk. Not only can they contribute to hepatotoxicity, but the altered drug metabolism in liver diseases may also lead to unpredictable pharmacokinetics, complicating dosing regimens and increasing the risk of adverse effects.

The intricate interplay between liver and kidney function adds a layer of complexity to pain management. NSAIDs, notorious for their potential to induce renal impairment, pose a dual threat in liver patients who may already grapple with compromised renal function [15]. The delicate balance between maintaining adequate analgesia and preventing further renal damage necessitates vigilant monitoring and individualized approaches. Gastrointestinal complications, including bleeding, represent a significant risk associated with NSAID use. In liver patients who may already contend with portal hypertension and varices, the potential exacerbation of gastrointestinal bleeding amplifies the complexity of pain management decisions [15]. Striking a balance between pain relief and the risk of bleeding requires a meticulous evaluation of each patient's clinical status and potential contraindications. Opioids, while providing effective pain relief, introduce a spectrum of central nervous system effects that demand careful consideration. Sedation, respiratory depression, and the risk of opioid-induced hyperalgesia add layers of complexity to opioid use in liver patients [16]. The challenge lies in optimizing pain control while minimizing the potential for adverse effects, striking a delicate balance that requires ongoing monitoring and dose adjustments.

Emerging Pharmaceutical Approaches for Liver-Related Pain

The limitations and potential risks associated with standard medications underscore the need for innovative approaches in reshaping the landscape of pain management for liver patients. Emerging pharmaceutical strategies offer glimpses into the future, holding the potential to address pain with greater precision and reduced adverse effects. Novel pharmaceutical approaches explore the intricacies of peripheral pain mechanisms, aiming to minimize systemic effects. Drugs that selectively target peripheral nociceptors or modulate neurotransmitter release at the injury site represent a paradigm shift. Still, in the early stages of development, such approaches hold promise in providing localized pain relief without imposing undue stress on the liver or other organ systems [16]. The era of precision medicine heralds a new frontier in pain management. Tailoring interventions based on an individual's genetic makeup, metabolic profile, and specific pain mechanisms allows for a more refined and personalized approach [17]. The application of pharmacogenomics, in which genetic information guides medication selection and dosing, holds the potential to minimize adverse effects and optimize pain control for liver patients. Cannabinoids, compounds derived from the cannabis plant, are gaining attention for their potential in pain management. Cannabidiol (CBD) and tetrahydrocannabinol (THC), two prominent cannabinoids, exhibit analgesic properties and anti-inflammatory effects. While the potential hepatotoxicity of cannabinoids remains a subject of debate, preliminary evidence suggests a role in managing neuropathic pain, especially in conditions like cirrhosis [17]. Neurostimulation techniques, such as spinal cord and peripheral nerve stimulation, offer an alternative avenue for pain management. By modulating pain signals at the neural level, these interventions aim to disrupt the pain pathway without relying on systemic medications [17]. While still considered investigational, neurostimulation holds promise as a potential option for liver patients resistant to or intolerant of traditional analgesics.

Non-pharmacological approaches

CLDs have a widespread impact, and among the complex array of difficulties, pain emerges as a formidable opponent. This essay explores non-pharmacological methods for managing pain in individuals with chronic liver illnesses. We uncover a story beyond traditional pain management methods by examining the impact of lifestyle changes, psychological treatments, and integrative therapies. This approach promotes overall well-being for individuals dealing with liver-related pain.

Role of Lifestyle Modifications in Pain Management

Lifestyle alterations, which are often overlooked in the field of pain management, have the power to bring about significant changes for persons dealing with chronic liver disorders. These alterations go beyond the conventional limits of medical therapy, embracing elements of daily life that profoundly impact the pain experience. In the setting of liver illnesses, diet plays a crucial role in managing pain. Dietary choices are essential for reducing inflammation, treating nutritional shortages, and promoting optimal liver function. Consuming a diet abundant in antioxidants omega-3 fatty acids, and low in processed carbohydrates may decrease inflammatory indicators, which could help relieve pain [18]. In addition, for persons with liver problems, maintaining an ideal body weight is not just a matter of appearance but a deliberate method to reduce the mechanical stress on the liver capsule. Excessive weight leads to heightened pressure on the liver, which can worsen pain symptoms. Thus, incorporating a well-balanced and hepatoprotective diet is crucial to pain therapy.

The interdependent connection between engaging in physical activity and effectively managing pain serves as a promising prospect for persons suffering from chronic liver disorders. Although exercise may first cause concern when considering damaged health, customized physical activity can provide many advantages. Scientific evidence has demonstrated that regular physical activity can decrease inflammation, promote cardiovascular health, and enhance general well-being [18]. Individuals suffering from chronic liver disorders might alleviate discomfort and improve their sense of empowerment and resilience by participating in activities such as walking, swimming, or mild yoga. However, it is vital to customize exercise routines to match the individual's health condition and restrictions. By fostering collaboration between healthcare doctors and fitness professionals, physical exercise can be transformed into a therapeutic asset rather than a burden. Within the field of pain management, the sometimes-disregarded element of sleep hygiene appears as a fundamental feature for patients suffering from chronic liver disorders. Insomnia, which is prevalent among individuals with liver conditions, not only intensifies the experience of pain but also contributes to a cycle of tiredness and reduced ability to recover. Implementing techniques to enhance sleep quality, such as adhering to a regular sleep routine, establishing a sleep-friendly atmosphere, and reducing the consumption of stimulating substances before bedtime, can significantly improve the rejuvenating nature of sleep. An adequately rested body is more capable of managing pain and participating in the necessary healing processes for persons dealing with CLDs [19].

Psychological Strategies for Managing Pain

The complex interaction between the mind and the perception of pain reveals a domain where psychological interventions arise as potent strategies for managing pain. These interventions go beyond the field of pharmacology and address the emotional and cognitive aspects of pain, providing patients with a sophisticated method for controlling the intricacies of their pain experience. Cognitive-behavioral therapy (CBT) is highly regarded as an effective psychological strategy for managing pain. Based on the premise that thoughts, feelings, and behaviors are interrelated, CBT seeks to reframe unhealthy thought patterns and develop effective coping strategies. CBT is a systematic approach to help people with CLDs who experience both physical pain and emotional suffering. It focuses on identifying and changing negative thought patterns and promoting resilience. CBT helps individuals by providing them with mental tools and methods to cope with pain. This reduces the psychological impact of pain and leads to noticeable improvements in how pain is perceived [19].

Mindfulness meditation, an old practice rooted in contemplative traditions, is now recognized as a valuable tool for managing pain in modern times. Mindfulness encourages individuals to develop conscious awareness in the present moment, promoting a non-evaluative acceptance of ideas and experiences. Mindfulness meditation provides solace for individuals grappling with CLDs, where pain frequently becomes an enduring presence. Studies have shown that mindfulness-based therapies can enhance pain intensity, how pain affects daily activities, and general well-being [19]. By embracing the current moment, individuals can effectively handle the fluctuation of pain with tranquility and acceptance. Pain frequently manifests itself through muscle tension and elevated stress levels. Engaging in various relaxation techniques, such as progressive muscle relaxation and guided imagery, can be a beneficial therapeutic pursuit for persons looking to alleviate liver-related pain. These techniques mitigate the tension knots often accompanying chronic pain, relieving physical and mental strain. Incorporating relaxation practices into everyday routines equips individuals with practical strategies for pain management, empowering them to take control of their path [20].

Exploring the Advantages of Integrative Therapies

Pain management encompasses more than traditional medical and psychological treatments, encouraging consumers to investigate integrative therapy. These therapies, frequently based on holistic traditions, enhance the current approaches by providing a diverse range of possible advantages. Acupuncture, a time-honored technique based on traditional Chinese medicine, is the precise insertion of slender needles into specific spots on the body. Acupuncture provides a distinct approach to addressing the complexities of energy pathways in the management of pain for persons with CLDs. Studies indicate that acupuncture can alleviate pain by regulating the release of neurotransmitters and impacting the body's perception of pain [20]. Acupuncture offers a comprehensive approach to pain management by targeting physical and energetic aspects, aligning with an individual's desire for complete alleviation. Massage treatment goes beyond relaxation and plays a role in managing discomfort for people suffering from CLDs. Engaging in tactile stimulation and manipulation of soft tissues helps relieve muscle tension and promotes the release of endorphins, which are the body's natural painkillers [20]. Integrating massage therapy into the pain treatment regimen provides clients with a concrete encounter of therapeutic touch. In addition to the physical advantages, the emotional and psychological aspects of pain can be alleviated by the comforting touch of a proficient massage therapist.

Individuals with CLDs are encouraged to consider herbal supplements as a complementary option to conventional interventions to benefit from nature's pharmacy. Although the scientific evidence about the effectiveness of herbal supplements may differ, specific herbs demonstrate anti-inflammatory and analgesic characteristics. Turmeric, containing the active component curcumin, shows the potential to reduce inflammation and offer analgesic effects. Likewise, ginger, renowned for its anti-inflammatory features, is a tasty inclusion in the collection of natural therapies [21]. Nevertheless, it is imperative to exercise prudence while dealing with herbal supplements, taking into account the possibility of conflicts with pharmaceuticals and seeking advice from healthcare professionals.

Precision medicine in pain management

Chronic pain, an often experienced condition in health difficulties, requires a detailed and individualized approach. Recently, the idea of precision medicine has gained attention as a promising approach to customizing pain management techniques based on the distinct attributes of each person. This essay aims to examine precision medicine in pain management thoroughly. It provides an overview of precision medicine concepts, explores the complexities of tailoring strategies to individual patient profiles, and discusses the advancements in pharmacogenomics and their significant implications.

Overview of Precision Medicine Concepts

Precision medicine aims to transcend the uniform approach by recognizing the intrinsic variations among individuals regarding their genetic composition, lifestyle, and environmental factors. Precision medicine seeks to customize therapies by considering the unique characteristics of individual patients instead of using standardized treatment approaches. The core principle of precision medicine is recognizing that our genetic composition significantly impacts how our bodies react to drugs and therapies. The Human Genome Project, a significant undertaking that fully mapped the complete human genome, established the basis for understanding the complex relationship between genetics and health [21]. Precision medicine utilizes genomic data to identify individual variances in drug metabolism, effectiveness, and probable adverse reactions. Healthcare professionals can customize interventions based on the distinct genetic profile of each patient, ensuring that the treatments are aligned with the individual's specific biological characteristics. Genomics is a fundamental aspect of precision medicine, but a comprehensive approach goes beyond genetics to include various elements that influence an individual's health profile. Lifestyle choices, environmental exposures, and socio-economic characteristics collectively affect the foundation on which precision medicine implements its customized interventions. In pain management, adopting a holistic viewpoint is of utmost importance. Pain is not solely a genetic manifestation; it is interconnected with psychological, social, and environmental aspects. Precision medicine in pain treatment entails a thorough evaluation that considers the genetic predispositions and the numerous circumstances that impact an individual's pain perception [21].

Tailoring Pain Management Strategies Based on Individual Patient Profiles

Precision medicine emerges as a revolutionary framework in pain management, enabling healthcare providers to surpass the conventional trial-and-error method in treatment. Customizing pain management strategies according to specific patient profiles requires thoroughly comprehending the patient's distinct attributes and utilizing this knowledge to develop precise therapies. An essential principle of precision medicine in pain management is acknowledging that pain problems are not uniform. Different subcategories of pain syndromes may fall within a larger category, each with its own unique underlying causes and reactions to therapies. Within the context of chronic pain, there are many subtypes, such as neuropathic pain, inflammatory pain, and nociceptive pain. Each category requires a customized strategy [22]. Precision medicine enables the classification of pain problems according to their underlying mechanisms, facilitating the development of focused therapies targeting the fundamental causes. Conventional pain management methods often consist of a systematic increase in treatments, where drugs and therapies are administered uniformly to a wide range of patients. Precision medicine challenges this established model by promoting personalized treatment strategies that consider each patient's unique attributes. When dealing with chronic pain, it may be necessary to customize the selection of medications according to the individual's genetic tendency for drug metabolism.

Pharmacogenomic testing, which analyzes the impact of an individual's genes on their reaction to drugs, is a significant tool in determining the choice of analgesic agents [22]. Furthermore, non-pharmacological interventions, such as physical or CBT, can be customized to match the patient's inclinations, way of life, and psychosocial circumstances. Precision medicine in pain management surpasses the fixed character of treatment programs, acknowledging that people may undergo variations in their pain profiles over time. Wearable technologies and mobile health applications enable real-time monitoring, which allows for the adjustment of interventions according to the changing needs of the patient [23]. For example, a patient suffering from persistent pain may employ a mobile application to monitor pain intensity, physical exertion, and sleep cycles. By utilizing this up-to-the-minute data, healthcare providers can obtain valuable information about the factors contributing to the worsening of pain and customize interventions accordingly. If sleep disruptions become a notable factor in causing pain, the therapeutic approach may shift toward addressing sleep hygiene and employing relaxing techniques.

Advances in Pharmacogenomics and their Implications

Pharmacogenomics, which investigates the genetic elements impacting drug reactions, is a notable advancement in precision medicine. Pharmacogenomics reveals how drugs can be chosen and how dosages can be improved for pain treatment by considering an individual's genetic characteristics. The metabolism of drugs, an intricate interaction of enzyme systems within the body, differs among individuals due to genetic variances. Pharmacogenomic testing examines crucial genes that produce enzymes involved in drug metabolism, providing insights into an individual's potential reaction to particular drugs. One example is the cytochrome P450 family of enzymes, including CYP2D6 and CYP3A4, vital in breaking down certain pain-relieving drugs, such as opioids [24]. Genetic differences in these enzymes can lead to individuals being classified as poor metabolizers, extensive metabolizers, or ultra-rapid metabolizers. Every category has an impact on how the body metabolizes drugs, affecting both their effectiveness and the likelihood of experiencing negative side effects.

The opioid crisis has highlighted the crucial necessity for implementing safer methods of prescribing in the field of pain management. Pharmacogenomics aids in understanding an individual's reaction to opioids, thereby contributing to this urgent matter. Genetic differences in opioid receptors, namely the mu-opioid receptor (OPRM1), have an impact on the effectiveness and possible adverse reactions of opioids [24]. Through the identification of genetic markers linked to heightened sensitivity or reduced response to opioids, healthcare providers can make well-informed choices regarding opioid prescriptions, thereby reducing the chances of overdose or insufficient pain management. The merging of pharmacogenomics and precision medicine facilitates the advancement of personalized analgesic treatment plans. Healthcare providers can utilize genetic information to tailor the selection of analgesic drugs, doses, and administration methods instead of using a standardized strategy for pain management. Recent research has investigated the application of pharmacogenomic testing to enhance pain management for illnesses such as osteoarthritis and postoperative pain [24]. Research indicates that integrating pharmacogenomics into pain management regimens not only enhances pain outcomes but also decreases the occurrence of adverse medication reactions.

Although the potential of precision medicine in pain management is undoubtedly intriguing, significant ethical concerns need to be addressed. Ensuring the responsible and fair incorporation of precision medicine into clinical practice requires careful consideration of matters about consent, privacy, and accessibility. Precision medicine entails examining genetic data, so obtaining informed consent becomes very important. Patients must receive comprehensive information regarding the ramifications of genetic testing, encompassing the potential identification of non-pain-related ailments and the psychological and social consequences of genetic data. The intricate nature of genomic data introduces additional elements to obtaining informed consent, requiring explicit communication of the inherent unpredictability associated with genetic predictions. When dealing with the genomic landscape of precision medicine, it is crucial to address patient concerns, ensure understanding, and respect autonomy [25]. The abundance of genetic data produced by precision medicine gives rise to worries regarding privacy and data security. Genomic data is intrinsically sensitive, as it includes information about the individual and their biological relatives. Implementing strong procedures for encrypting, storing, and transmitting data is crucial to protect against unwanted access and potential misuse. Furthermore, the possibility of unforeseen outcomes, such as genetic discrimination by insurance companies or employers, emphasizes the necessity of legal and ethical structures that safeguard individuals from improper use of their genetic data [25].

The potential of precision medicine in pain management should not be limited to select segments of the population. It is essential to responsibly adopt measures that guarantee fair access to genetic testing and personalized interventions. To tackle inequalities in access, focusing on socio-economic variables, geographical issues, and cultural subtleties is necessary. Incorporating precision medicine into pain management requires proactive efforts to eliminate obstacles that may contribute to ongoing health disparities [25]. The practice of precision medicine in pain management prioritizes the involvement of patients in the decision-making process. Providing patients with information about the possible advantages and restrictions of genetic testing promotes a setting where well-informed decisions can be made. Efforts to educate patients, openly discuss the consequences of genetic information, and engage in joint decision-making foster a collaborative relationship between healthcare practitioners and patients. Focusing on precision medicine empowers patients to actively engage in their pain treatment journey, thereby contributing to a collaborative decision-making process [26].

Interdisciplinary collaboration

CLDs have a widespread impact, and among the complex array of difficulties, pain poses a significant and demanding problem. Acknowledging the complex and varied nature of pain experienced by liver patients emphasizes the necessity for collaboration between different disciplines. Hepatologists and pain experts, with their distinct areas of expertise, come together to form a collaborative partnership focused on understanding the intricacies of liver-related pain. In the field of liver diseases, where pain frequently intersects with the complexities of inflammation, fibrosis, and comorbidities, the partnership between hepatologists and pain experts becomes crucial [26]. Hepatologists, equipped with the knowledge of fundamental liver disorders, aid in identifying pain triggers and developing precise treatment strategies. However, pain specialists with extensive expertise in the intricacies of pain treatment possess a wide range of interventions that go beyond the conventional use of medications.

Utilizing Collaborative Methods for Holistic Pain Management

To effectively manage pain in liver patients, it is crucial to move away from the fragmented approach to healthcare and instead coordinate a holistic pain care plan. The emergence of team-based methods involving the collaboration of hepatologists, pain specialists, nurses, psychiatrists, and physical therapists represents a paradigm change surpassing individual expertise's constraints. As primary healthcare professionals, nurses connect the hepatology and pain management fields. Their everyday encounters with patients offer a distinctive perspective for evaluating the comprehensive influence of pain on persons' lives. Nurses are crucial in ensuring ongoing care and support for liver patients experiencing discomfort. They achieve this by educating patients, monitoring their symptoms, and facilitating effective communication across different medical specialties [27]. The psychological aspects of pain experienced by liver patients necessitate the involvement of psychologists with specialized knowledge. Psychologists work closely with hepatologists and pain experts to explore the emotional aspects of patients' experiences, focusing on anxiety, sadness, and the effects of pain on their overall well-being. Incorporating psychological therapies, such as cognitive-behavioral therapy, becomes a fundamental aspect of the comprehensive care framework. Physical therapists have a complete awareness of the physical symptoms associated with liver-related discomfort, in addition to their expertise in rehabilitative exercises. Physical therapists work closely with hepatologists and pain specialists to design customized exercise programs that relieve pain and promote functional recovery. Their proficiency in managing musculoskeletal conditions and enhancing mobility adds to a comprehensive approach to pain management [27].

Improving Communication and Collaboration Among Healthcare Practitioners

Effective communication and coordination among healthcare providers is the foundation for successful interdisciplinary collaboration. Facilitating open communication, collaborative decision-making, and prioritizing the patient's needs is crucial for creating a harmonious relationship among different areas of expertise. Regular interdisciplinary meetings, where hepatologists, pain specialists, and allied healthcare providers gather, function as a central point for collaboration. These meetings serve as a forum for analyzing cases, exchanging perspectives, and making decisions together. By engaging in a collaborative process of generating ideas, healthcare providers can enhance treatment plans, tackle difficulties, and coordinate actions to maximize pain management for patients with liver conditions [28].

Electronic health records (EHR) integration serves as a technology infrastructure that optimizes the transmission of information between healthcare practitioners. Enabling hepatologists and pain experts to access patient records collectively guarantees a thorough comprehension of the patient's medical history, liver condition, and pain management treatments. The instantaneous transmission of information reduces communication gaps and improves the consistency of healthcare delivery. Adopting a patient-centered approach, which involves individuals actively engaging in decision-making and goal-setting, catalyzes improving communication. By working together to create care plans that align with the patient's preferences, values, and lifestyle, we can ensure that the interventions are well-suited to the individual's specific requirements [28]. Enabling patients to participate in their pain management process actively enhances their sense of control and encourages them to follow the entire care plan. Interdisciplinary collaboration thrives when healthcare practitioners possess a shared comprehension of one another's roles, knowledge, and viewpoints. Interprofessional education programs involving hepatologists, pain specialists, and other team members are crucial in promoting a culture of mutual respect and recognition for diverse contributions through cross-disciplinary learning. Workshops, seminars, and collaborative training programs help dismantle professional barriers and foster a culture of collaboration.

Future directions and opportunities: paving the path for transformative pain management

The field of pain management for liver patients is currently on the verge of significant advancements, as continuing research is uncovering new knowledge and potential revolutionary discoveries. The convergence of hepatology, pain management, and state-of-the-art research provides opportunities for pioneering approaches that target the unique difficulties presented by liver-related pain. Ongoing research investigates the complex relationship between neuroinflammation and pain in the context of CLDs. Gaining insight into the role of inflammation in the central nervous system in pain perception reveals new possibilities for focused therapies. Manipulating neuroinflammatory pathways using drugs or immunomodulatory methods can reduce pain at its origin [28]. Advanced neural imaging techniques, such as functional MRI (fMRI) and positron emission tomography (PET), provide insight into the pain circuits in the brain. Research efforts on the neurological basis of liver-related pain seek to decipher the complex signals and reactions that contribute to the experience of pain. Neuroimaging research in this field offers a detailed comprehension of how the brain contributes to the perception of pain, which allows for the development of precise therapies that can disrupt dysfunctional neural circuits [28].

Possible Advancements in Pain Control for Individuals with Liver Conditions

Immunomodulatory medications, initially developed for illnesses including rheumatoid arthritis and inflammatory bowel disease, are now becoming recognized as potentially transformative treatments for liver-related pain. These therapies, which focus on specific elements of the immune system, show potential in adjusting the inflammatory environment contributing to pain in CLDs [29]. The ongoing clinical trials are investigating the safety and effectiveness of immunomodulatory drugs. This could lead to a redefinition of how inflammation is managed in cases of liver-related pain. The emerging discipline of gene therapy provides a genetic toolbox for precise pain alleviation in individuals with liver conditions. Gene therapy utilizes viral vectors to transport therapeutic genes to regulate the expression of target molecules that play a role in pain pathways [30]. Preclinical investigations on the efficacy of gene therapy in animal models of CLDs demonstrate initial achievements, sparking hope for a future where genetic therapies serve as precise instruments in pain management [31-33].

Significance of Ongoing Clinical Trials and Studies

Clinical trials and research studies are the arena for testing new therapies and therapeutic approaches. Continuing efforts have far-reaching consequences that transcend beyond research facilities and medical centers, influencing pain management for individuals with liver conditions. Peripheral nerve therapies in clinical trials provide opportunities to explore pain transmission routes separate from the central nervous system [33-36]. Peripheral nerve blocks, radiofrequency ablation, and neuromodulation procedures interrupt pain signals before they arrive at the spinal cord and brain. Continuing research examining the safety and effectiveness of these treatments in individuals with liver conditions offers valuable knowledge about their potential contribution to the comprehensive pain management approach [29].

Incorporating PROs into clinical trials is a significant change that enhances the role of the patient in assessing the effectiveness of pain management therapies [37-39]. PROs offer a comprehensive perspective for evaluating the effectiveness of interventions by considering the patient's subjective experiences, pain levels, and quality of life. Continuing research that utilizes PROs helps to develop a detailed understanding of the patient's viewpoint. This knowledge is then used to improve therapies to match the individual's priorities better [40]. Amidst a period characterized by technological progress, it is essential to conduct continuous research to examine the effectiveness of telehealth interventions in alleviating pain for individuals with liver conditions. This research is vital for addressing disparities in healthcare accessibility. Telehealth services, which include virtual consultations, remote monitoring, and digital interventions, provide a crucial solution for persons who encounter obstacles in accessing conventional healthcare. The ongoing trials evaluate telemedicine's possibility and efficacy in managing liver-related pain. These trials reveal telehealth's potential benefits in improving access to comprehensive care.

## Conclusions

To summarize, our investigation into customized pain control for individuals with liver conditions reveals a complex environment filled with difficulties and possibilities, emphasizing the delicate relationship between pain and chronic liver ailments. The various challenges, which include the complex causes of liver-related pain, the complexities of having multiple health conditions, and how medications interact with each other, emphasize the urgent need for a fundamental change in how we approach clinical practices. In the face of these difficulties, the possibilities that arise from interdisciplinary cooperation, precision medicine, and continuous research indicate a potential for significant change in comprehensive pain management. A resounding plea for action echoes throughout the research and clinical practice arenas, advocating a unified dedication to enhancing customized therapies for patients with liver conditions. This call to action is founded upon the amalgamation of hepatologists' understanding of liver diseases, pain specialists' proficiency in intricate pain management, and the prospective advancements derived from ongoing research. As we explore this field, driven by empathy and creativity, the future of pain treatment for liver patients urges us to adopt customized approaches that relieve symptoms and reframe the story of strength and self-empowerment. Our shared dedication creates a path toward a future where personalized and empathetic care for liver-related pain goes beyond traditional boundaries and strives for holistic wellness.
